# Randomized Trial of Glucosamine and Chondroitin Supplementation on Inflammation and Oxidative Stress Biomarkers and Plasma Proteomics Profiles in Healthy Humans

**DOI:** 10.1371/journal.pone.0117534

**Published:** 2015-02-26

**Authors:** Sandi L. Navarro, Emily White, Elizabeth D. Kantor, Yuzheng Zhang, Junghyun Rho, Xiaoling Song, Ginger L. Milne, Paul D. Lampe, Johanna W. Lampe

**Affiliations:** 1 Division of Public Health Sciences, Fred Hutchinson Cancer Research Center, Seattle, Washington, United States of America; 2 Department of Epidemiology, Harvard School of Public Health, Boston, Massachusetts, United States of America; 3 Division of Clinical Pharmacology, Vanderbilt University, School of Medicine, Nashville, Tennessee, United States of America; Indiana University Richard M. Fairbanks School of Public Health, UNITED STATES

## Abstract

**Background:**

Glucosamine and chondroitin are popular non-vitamin dietary supplements used for osteoarthritis. Long-term use is associated with lower incidence of colorectal and lung cancers and with lower mortality; however, the mechanism underlying these observations is unknown. *In vitro* and animal studies show that glucosamine and chondroitin inhibit NF-kB, a central mediator of inflammation, but no definitive trials have been done in healthy humans.

**Methods:**

We conducted a randomized, double-blind, placebo-controlled, cross-over study to assess the effects of glucosamine hydrochloride (1500 mg/d) plus chondroitin sulfate (1200 mg/d) for 28 days compared to placebo in 18 (9 men, 9 women) healthy, overweight (body mass index 25.0–32.5 kg/m^2^) adults, aged 20–55 y. We examined 4 serum inflammatory biomarkers: C-reactive protein (CRP), interleukin 6, and soluble tumor necrosis factor receptors I and II; a urinary inflammation biomarker: prostaglandin E2-metabolite; and a urinary oxidative stress biomarker: F_2_-isoprostane. Plasma proteomics on an antibody array was performed to explore other pathways modulated by glucosamine and chondroitin.

**Results:**

Serum CRP concentrations were 23% lower after glucosamine and chondroitin compared to placebo (*P* = 0.048). There were no significant differences in other biomarkers. In the proteomics analyses, several pathways were significantly different between the interventions after Bonferroni correction, the most significant being a reduction in the “cytokine activity” pathway (*P* = 2.6 x 10^-16^), after glucosamine and chondroitin compared to placebo.

**Conclusion:**

Glucosamine and chondroitin supplementation may lower systemic inflammation and alter other pathways in healthy, overweight individuals. This study adds evidence for potential mechanisms supporting epidemiologic findings that glucosamine and chondroitin are associated with reduced risk of lung and colorectal cancer.

**Trial Registration:**

ClinicalTrials.gov NCT01682694

## Introduction

Glucosamine and chondroitin (G&C), often taken together as a single pill and used for osteoarthritis, are among the most popular dietary supplements in the US [1]. G&C are considered safe, with no known major adverse side effects [2]. While the efficacy of these supplements for treatment of osteoarthritis remains debated [2,3], recent studies suggest that they may have potential for reducing risk of other chronic diseases, such as cancer. In the VITamins And Lifestyle (VITAL) study, a large (n = 77,738) prospective cohort, we reported that use of G&C supplements is associated with a 27–35% lower incidence of colorectal cancer [4], a 26–28% lower incidence of lung cancer [4], 17% lower overall mortality [5], and a 13% lower cancer mortality [6]. These findings on G&C were the most consistent results across endpoints among the 30 supplements that were the focus of the VITAL study.

It is generally well-accepted that chronic inflammation contributes to carcinogenesis [7]. The majority of cellular events involved in the inflammation process require nuclear factor kappa B (NK-κB), a transcription factor that plays a central role in the generation of cytokines, chemokines and other soluble factors involved in the immune response. Similarly, excess reactive species characteristic of oxidative stress can damage cell membranes and DNA [8], and are thought to contribute to genomic instability and development of cancer [9]. Several lines of compelling evidence from *in vitro* and preclinical studies support a possible role for G&C in reducing inflammation [10–13]. A limited number of human observational and randomized studies also suggest that G&C lower systemic biomarkers of inflammation and oxidative stress [14,15]; however, no human intervention trials have evaluated the potential for G&C to reduce inflammation or alter other pathways relevant to carcinogenesis in healthy individuals free of chronic inflammatory conditions.

Our objectives in the present study were two-fold. Our first aim was to determine the effect of a common dose of G&C on a panel of serum inflammation biomarkers [high sensitivity C-reactive protein (CRP), interleukin-6 (IL-6), and soluble tumor necrosis factor receptors I & II (sTNFRI and II); a urinary biomarker of inflammation, prostaglandin E_2_-metabolite (PGE-M); and a urinary biomarker of oxidative stress, F_2_-isoprostane (F_2_-IsoP)]. Secondly, we sought to characterize intervention-induced changes in plasma proteomic patterns via pathway analyses in response to G&C compared to placebo. Results from this study may help determine the anti-inflammatory potential of G&C in humans, and may identify other possible pathways that may be modulated with use of G&C.

## Methods

### Research Design

The study was a randomized, double-blind, placebo-controlled, crossover trial comparing supplemental glucosamine and chondroitin to placebo. The protocol for this trial and supporting CONSORT checklist are available as supporting information; see S1 Protocol and S1 CONSORT Checklist. As this was a small pilot trial with the aim of enrolling a sample of 20 individuals, participants were randomly assigned by the project manager to begin with either the active or placebo intervention period in blocks of two using a pre-determined randomization template alternating placebo and G&C interventions. Each new recruit was assigned to the next available assignment. Each intervention period lasted 28 days with a 28 day washout period between the two interventions. All study activities were carried out at the Fred Hutchinson Cancer Research Center (FHCRC), Seattle, Washington. Recruitment, enrollment, trial, and sample collection took place from October 2012 to July 2013. The study protocol was approved by the FHCRC Institutional Review Board on July 11, 2012, with continuation approval obtained through May 18, 2015. All participants gave informed written consent. The authors confirm that all ongoing and related trials for this intervention are registered. This trial was registered at http://www.clinicaltrials.gov as NCT01682694.

### Participants

Participants were healthy, overweight [BMI (body mass index) >25 and ≤32.5 kg/m^2^], non-smoking, aged 20–55 years, and recruited from the greater Seattle area. We recruited men and women who were overweight because inflammation biomarkers are unlikely to decrease measurably in response to an intervention among individuals who have very low systemic inflammation at baseline, and BMI is a strong predictor of both CRP and IL-6 [16,17]. Methods for recruitment included flyers posted on university campuses in Seattle and at FHCRC, an informational website, and advertisements in campus and local neighborhood newspapers.

Screening occurred in two phases. In the first phase, individuals were screened for eligibility using a self-administered questionnaire and were excluded for any of the following: chronic medical illness; history of gastrointestinal, hepatic, or renal disorders; inflammatory conditions (including autoimmune and inflammatory diseases); pregnancy or lactation; currently on a weight loss diet; BMI <25 or ≥32.5 kg/m^2^; alcohol intake >2 drinks/day (2 drinks defined as 720 ml beer, 240 ml wine or 90 ml spirits); current use of prescription or over-the-counter medications other than oral contraceptives and hormone-secreting IUDs, multivitamins or use of aspirin or NSAIDs more than 2 days per week; inability to swallow pills; known allergy to shellfish; not willing to take pills made from shellfish or animal sources; or intention to relocate out of the study area within the next 4 months. Eligible individuals were invited to a study information session and those interested in participating provided informed written consent.

In the second phase of screening, prospective participants attended a screening clinic visit at the FHCRC Prevention Center. Height and weight were measured to ensure BMI requirements were met. Because body fat can vary among similar BMIs, body fat percentage was assessed by whole-body dual-energy X-ray absorptiometry (DEXA) scanning using a GE Lunar DPX-Pro densitometer (GE Healthcare, Milwaukee, WI). Blood was drawn in the morning after an overnight fast and was used for analysis of renal (blood urea nitrogen: 7–25 mg/dl; creatinine: 0.50–1.10 mg/dl for females; 0.60–1.35 mg/dl for males; eGFR ≥ 60 ml/min/1.73m^2^; sodium: 135–146 μmol/l; potassium: 3.5–5.3 μmol/l; chloride: 98–110 μmol/l), liver (albumin: 3.6–5.1 g/dl; globulin: 2.1–3.7; total bilirubin: 0.2–1.2 mg/dl; alkaline phosphatase: 40–115 U/L; AST: 10–30 U/L for females; 10–40 U/L for males; ALT: 6–40 U/L for females; 9–60 U/L for males), and metabolic function (glucose: 65–99 mg/dl). Individuals with normal laboratory values were invited to participate in the study.

**Fig 1:**
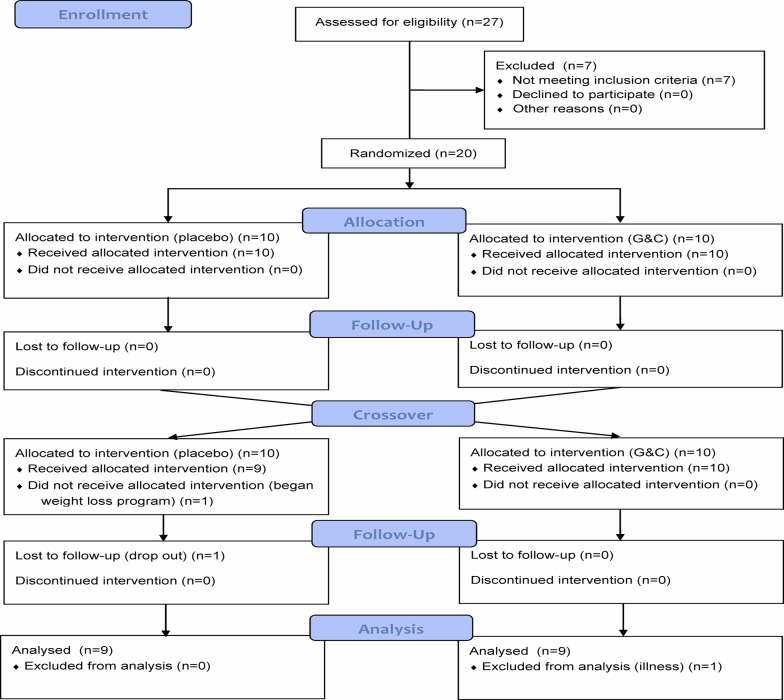
CONSORT Flow diagram of participants in the glucosamine and chondroitin (G&C) randomized, placebo-controlled trial.

A total of 27 individuals underwent screening activities. Seven were ineligible prior to randomization, six due to high fasting glucose concentrations and one due to abnormal liver enzyme concentrations. Twenty individuals (n = 10 men; n = 10 women) were randomized. One female was asked to drop out after completing only one intervention period because she started a new diet and exercise program that might have had effects on biomarker measures. Because inflammation biomarkers are influenced by minor infections (e.g., colds) and other illnesses [18], participants were instructed to delay clinic visits until they were no longer ill; however, one male participant attended a clinic visit at the onset of a cold and was also excluded, leaving a sample size of 18 (9 men and 9 women; Fig. 1).

### Glucosamine and Chondroitin Supplements

Our aim was to evaluate G&C as used in population settings. Thus, dosing was based on the common starting dose recommended by the product manufacturer, which is similar across G&C products. The active treatment was 1500 mg/d FCHG49 glucosamine hydrochloride (GHCl) + 1200 mg/d TRH122 sodium chondroitin sulfate (CS) taken as 3 capsules daily with each capsule containing 500 mg GHCl and 400 CS. Crystalline cellulose was used as an inactive filler, and both active treatment and placebo were encapsulated in clear gelatin capsules. The placebo was similar in appearance and contained only the inactive filler. Both G&C and the placebo were generated from a single lot of source materials and donated by Nutramax Laboratories Consumer Care, Inc. (Edgewood, MD). Supplement bottles were provided by the manufacturer with a single printed label containing the letters “A” or “B”. To maintain double-blinding for both participants and investigators, the randomization algorithm was sent in a sealed envelope to an investigator uninvolved in the day to day study activities; unblinding of the interventions was done at the time of data analysis. Participants were contacted by study staff at the mid-point and end of each intervention period to assess any possible adverse experiences and compliance. No adverse events were reported. Extra pills were included in each supplement bottle in the event that a Day 28 study visit was delayed, and so that adherence percentages (pills supplied—pills returned/days elapsed) could be calculated as an additional compliance measure. Composition of analysis testing was performed by an independent laboratory (Tampa Bay Analytical Research, Inc., Clearwater, FL) in collaboration with the NIH Office of Dietary Supplements. HPLC-UV analysis of a subset of capsules confirmed that mean G&C content was within specifications. No G&C was detected in the placebo capsules and no microbial products were detected in either capsule type.

### Specimen Collection

Biological samples were collected at baseline (screening visit) and after each 28-day intervention period in the morning after a minimum of a 12-hour overnight fast. For blood samples, tubes without additive were allowed to clot at room temperature for 30 minutes before they were centrifuged to separate the serum. Blood was also drawn into a tube containing EDTA for plasma. Both serum and plasma were aliquoted and stored at -80°C.

Participants were given a urine collection kit and instructions before each visit, and brought the specimen with them to the clinic visit. Participants were instructed to collect all of their urine for the 12 hours prior to their morning clinic visit at baseline and on Day 28 of each intervention period. Urine-void times were recorded and any uncollected voids noted. Urine specimens were stored at 4°C until delivery to the FHCRC the morning following collection. Urine volume was measured and aliquots stored at 80°C.

### Laboratory Analyses

#### Serum Inflammation Biomarkers

All assays were performed on never-thawed samples with the exception of CRP which is stable through freeze-thaw cycles, and was measured in samples that had been thawed once [19]. No observations were below the limit of detection for any markers. Assays for all inflammation markers have been described [20]. Intra and inter-assay CVs for CRP, IL-6, and sTNFRI and II were 1.7% and 5.5%; 3.7% and 5.8%; 2.1% and 1.7%; and 2.3% and 5.6%, respectively.

#### Urinary Inflammation and Oxidative Stress Biomarkers

Urinary PGE-M (11-alpha-hydroxy-9,15-dioxo-2,3,4,5-tetranor-prostane-1,20-dioic acid), the stable metabolite of PGE_2_, was assayed at the Vanderbilt Eicosanoid Core Laboratory (VECL, Nashville, TN) by a liquid chromatography/mass spectrometry (LC/MS) method, as previously described [21]. Urinary F_2_-IsoP was measured by gas-chromatography (GC)-negative-ion chemical ionization MS by the VECL using an Agilent 5973 GC/MS system (Santa Clara, CA) [22].

#### Proteomics Analysis of Plasma

Plasma samples were evaluated on a customized antibody array populated with ~3,000 full-length antibodies, printed in triplicate on a single microarray [23–25]. Briefly, to analyze proteins in the plasma samples, we depleted albumin and IgG. Protein (200 μg) from the two participant blood draws were each labeled with Cy5. Each sample was combined with reference sample and pipetted at the microarray/coverslip junction followed by incubation for 90 min at room temperature in the dark. Unbound proteins were removed by washing and the slides scanned for Cy3 and Cy5 fluorescence in an Axon Genepix 4000B scanner. The Cy5/Cy3 ratio determined the relative concentration of protein compared to reference. The array contains antibodies to cytokines (e.g., interleukins 1A, 1B, 2, 3, 4, 4R, 5, 6, 7, 8, 10, 12A, 13, 15, 16, 17D, 18, 19, 20, 24, 28A, and 34 and interleukin receptors 2RA, 2RA, 3RA, 4R, 5RA, 6ST, 17RA and 17RB), adipokines, proteins involved in apoptosis, angiogenesis, T-cell activation/infiltration, inflammation/prostaglandins, insulin, insulin resistance toll-like receptor (TLR), transforming growth factor (TGF)-β, and STAT signaling pathways that have been reported to be deregulated in a variety of diseases. Most (>85%) antibodies on the array had coefficients of variation, for triplicates, of less than 10% [23,24,26,27]. Detailed protocol descriptions of array fabrication, plasma treatment, plasma labeling, incubation, and scanning have been published elsewhere [23,27].

### Array Analysis and Normalization

Array data contain a format identical to two-channel gene expression arrays and analysis proceeds analogously. The array image was scanned using a GenePix 4000B (Axon Instruments) scanner. The numerical data processed by Genepix Pro 6.0 were imported to Limma 2.4.11 (Linear Models for Micro Array, a Bioconductor R package [28]). For each antibody, fold-change of the signal (red channel) was compared to the reference (green channel). The M value was calculated as Red/Green, where Red and Green were computed after background correction using the “normexp” method [29]. Saturated array spots were flagged and triplicate antibodies with coefficients of variation >10% were removed. Experimental variation was normalized using within-array print-tip loess and between-array quartile normalization. Triplicate features were summarized using their median. M values were standardized such that the mean value and standard deviation of the placebo group was set to zero and one, respectively. After all processing, data were available for analysis on a total of 2938 antibodies.

### Statistical Analysis

All inflammatory and oxidative stress biomarkers were log-transformed to improve the normality of the distributions prior to analysis. Results are presented as geometric means with 95% confidence intervals (CI). Generalized estimating equations (GEE), which account for correlation of repeated measures, were used to assess the effects of G&C versus placebo on inflammation and oxidative stress. Analysis of each Day 28 inflammatory or oxidative stress biomarker was adjusted for the same biomarker concentration at Day 0 for each treatment period. Sex, age, and body-fat percent were selected *a priori* as additional covariates for adjustment in the model. We also evaluated the effect of treatment period but because it did not affect the point estimates, (i.e., carryover effects were not contributing to outcome measures), we did not include treatment period in the models. The two-sided *P* value for statistical significance for biomarkers of inflammation and oxidative stress was set at <0.05. Analyses were performed using Stata statistical software (v13.1, StataCorp, College Station, TX).

Proteomics data were analyzed by performing paired *t*-tests to determine statistically significant differences between the two interventions, after adjusting for covariate effects, including hybridization day, and plate and box position by linear regression. Antibody markers were ranked on the basis of p value and effect sizes, so that a positive value indicates that the antibody expression was greater after supplementation with G&C compared to placebo, and a negative value indicates lower expression. To further integrate the antibody at the protein level, multiple duplicated antibodies for the same protein that did not have at least two significant results with the effect size going in the same direction were excluded. Among those proteins with at least two concordant significant results, the most significant antibody was reported for the top 100 proteins. Gene Set Enrichment Analyses, the evaluation of microarray data at the level of “gene groups” that share a common biologic theme or cellular location, were carried out based on the *t*-test results using Kyoto Encyclopedia of Genes and Genomes (KEGG) [30] and the gene set database Gene Ontology (GO) [31] downloaded from the Molecular Signatures Database (MSigDB, http://www.broadinstitute.org/gsea/msigdb/index.jsp). We used a Bonferroni correction to determine significant differences within the KEGG pathway [*P* <3.85x10^-4^; (0.05/130, the total number of pathways evaluated in KEGG)], the GO pathway [*P*<5.18x10^-5^; (e.g., 0.05/966, the total number of pathways evaluated in GO)], and for the individual proteins [*P* <1.70x10^-5^; (0.05/2,938, the total number of antibodies on the array)].

## Results

Table 1 summarizes the demographic and baseline characteristics of the 18 study participants stratified by sex. Baseline measures of all inflammatory biomarkers were higher among men, while F_2_-isoP was lower.

**Table 1:** Characteristics of participants randomized in the crossover trial of glucosamine and chondroitin at baseline (n = 18).

	Men (n = 9)	Women (n = 9)
Personal characteristics ^1^		
Age (y)	40 (8.0)	38 (8.8)
BMI (kg/m^2^)	27 (2.8)	29 (2.4)
Body fat (%)^2^	32 (4.6)	39 (4.1)
Inflammation biomarkers ^3^		
CRP mg/l	1.18 (1.3)	0.77 (1.1)
IL-6 pg/ml	1.19 (0.9)	0.52 (0.2)
sTNFRI pg/ml	1119 (287)	654 (398)
sTNFRII pg/ml	5832 (1280)	5398 (620)
PGE-M (ng/mg creatinine)	8.32 (4.7)	3.83 (1.3)
F_2_-isoprostane (ng/mg creatinine)	1.00 (0.4)	1.13 (0.4)

^1^Mean (SD)

^2^Measured by Dual X-ray Absorptiometry (DEXA)

^3^Geometric mean (geometric SD)

Statistically significantly lower geometric mean CRP concentrations were observed after the G&C intervention compared to placebo (-23%, *P* = 0.048; Table 2). No significant differences were observed in the other inflammation or oxidative stress biomarkers assessed, although they were slightly lower after G&C supplementation with the exception of the soluble TNF receptors, which increased by 1–3%.

**Table 2:** Inflammatory and oxidative stress biomarker concentrations after placebo and glucosamine and chondroitin intervention.

Biomarker	Placebo (N = 18)	Glucosamine & Chondroitin (N = 18)	*P*
	Mean (SD)^1^	Mean (SD)^1^	
CRP (mg/l)	1.17 (0.17)	0.90 (0.13)	0.048
IL-6 (pg/ml)	0.89 (0.10)	0.81 (0.09)	0.27
sTNFRI (pg/ml)	871.3 (15.6)	901.6 (15.7)	0.17
sTNFRII (pg/ml)	5558 (103)	5633 (104)	0.34
PGE-M (ng/mg creatinine)	6.15 (0.41)	5.89 (0.39)	0.60
F_2_-isoprostane (ng/mg creatinine)	1.20 (0.08)	1.10 (0.08)	0.38

^1^Least squares geometric means (geometric SD) from GEE model adjusted for baseline values, age, sex, and body fat percent

Of the ~3,000 antibodies on the proteomics array, 2,938 were detected. We conducted gene-set enrichment pathway analyses using both KEGG and GO database tools (Table 3). Five pathways in the KEGG pathway analysis remained statistically significant after a Bonferroni correction (nominal p-values 7 x 10^-10^ to 4 x 10^-5^)—cytokine-cytokine receptor interaction, JAK/STAT signaling pathway, intestinal immune network for IGA production, leukocyte transendothelial migration, and ubiquitin-mediated proteolysis. Twenty-five pathways in the GO pathway analyses remained statistically significant after Bonferroni correction (p values 3 x 10^-16^ to 1 x 10^-5^), including cytokine activity, receptor binding, hematopoietin/interferon (classD 200 domain) cytokine receptor binding, growth factor activity, extracellular region, chemokine activity, extracellular space, G-protein coupled receptor binding, chemokine receptor binding, extracellular region part, locomotory behavior, membrane fraction, response to external stimulus, regulation of actin filament length, regulation of actin polymerization and/or depolymerization, regulation of cellular component size, behavior, actin polymerization and/or depolymerization, regulation of organelle organization and biogenesis, regulation of cytoskeleton organization and biogenesis, response to biotic stimulus, nucleus, microtubule-based process, response to other organism and cell fraction. In each analysis, cytokine activity/cytokine receptor binding was the most significantly affected pathway. With the exception of ubiquitin-mediated proteolysis, and proteins located in the nucleus, all pathways were down-regulated after G&C supplementation.

**Table 3:** Significantly different pathways comparing glucosamine and chondroitin supplementation to placebo via gene-set enrichment analysis in Kyoto Encyclopedia of Genes and Genomes (KEGG) and Gene Ontology (GO) databases (n = 18).

Pathway/ Genes (count)^1^	Probes in Array^2^	Positive Probes^3^	Negative Probes^4^	*P* value*	Genes in the Pathway
***KEGG*^*5*^**					
Cytokine-Cytokine Receptor Interaction					
101	368	70	165	7.2x10^-12^	CSF3;IL13;IL8;EGFR;TNF;LIFR;CXCL12;PRLR;CCL20;IL4;IL6;IL10;CCL5;TNFRSF9;PRL;FLT4;IL5RA; CD40LG;IL28A;KIT;VEGFA;IL24;TNFRSF17;LEP;CD27;IFNGR1;IL15;IFNB1;CCL14;TNFRSF1B;IL20; IL15RA;FLT3;FASLG;IL18;CSF2;MPL;IL1A;IL1RAP;IL18RAP;PDGFRA;CSF1;LEPR;TNFRSF11B; TNFRSF10B;IL2RA;NGFR;IL10RB;FIGF;CXCL11;CSF3R;IFNG;TGFB1;CXCR4;PF4;CCR5;IL6ST; TGFBR2;IL3RA;CCL4;TGFB3;IFNAR1;IL12A;TNFSF8;PDGFRB;IL4R;MET;EGF;IL7;TGFB2;KDR;CXCL5;TNFSF13B;CCL27;IL5;FLT1;IL13RA1;CCR7;TNFSF9;CXCR3;BMPR1A;IL19;TNFRSF10A;TNFRSF8; FAS;INHBE;PDGFA;BMP7;INHBC;CCL21;CCR2;GHR;CCR6;PDGFB;VEGFB;OSM;CD70;CXCL10; IFNA1;IL12RB2;INHBA
JAK/STAT Signaling					
71	211	39	104	4.4x10^-8^	CSF3;IL13;LIFR;PRLR;IL4;IL6;IL10;CCND3;PRL;IL5RA;IL28A;IL24;LEP;IFNGR1;IL15;IFNB1;PIK3CB; STAT5A;CCND1;SOCS7;MYC;IL20;IL15RA;CSF2;MPL;PIM1;CBLB;LEPR;STAT1;IL2RA;IL10RB;CSF3R;IFNG;CSH1;GRB2;PIK3CA;IL6ST;IL3RA;IFNAR1;IL12A;AKT1;IL4R;IL7;PTPN6;STAT5B;PTPN11;IL5; STAT4;IL13RA1;BCL2L1;SOCS2;SOS1;STAT3;IL19;CCND2;SPRED1;AKT2;CBL;JAK3;TYK2;GHR; SOCS1;SPRY1;PIK3R1;CREBBP;OSM;PIK3CD;IRF9;CBLC;IFNA1;IL12RB2
Intestinal Immune Network for IGA Production					
15	88	17	51	8.7x10^-6^	CXCL12;IL4;IL6;IL10;CD40LG;CD86;TNFRSF17;IL15;IL15RA;TGFB1;CXCR4;CD28;TNFSF13B;CCL27; IL5
Leukocyte Transendothelial Migration					
38	109	19	53	3.5x10^-5^	CXCL12;CLDN18;ITGB1;PTK2B;RAC1;MMP9;PLCG1;PIK3CB;THY1;VCL;BCAR1;VASP;MMP2; VCAM1;ACTB;PECAM1;RAP1A;RHOA;PTK2;CXCR4;PIK3CA;ITGAL;ITK;GNAI3;PTPN11;VAV3;PXN; CDH5;CLDN6;RASSF5;PRKCA;PIK3R1;MAPK11;PIK3CD;CLDN1;ITGAM;SIPA1;MAPK14
Ubiquitin-Mediated Proteolysis					
23	37	19	3	4.1x10^-5^	BRCA1;MDM2;BIRC2;BIRC3;MAP3K1;CBLB;CUL4A;CUL2;CUL5;XIAP;CUL4B;CUL7;UBE2S;FANCL; CUL1;CBL;UBR5;SOCS1;ANAPC2;SKP2;VHL;FBXW7;CBLC
***GO*** ^***5***^					
Cytokine Activity					
41	177	23	109	2.6x10^-16^	CSF3;IL8;TNF;CXCL12;CCL20;IL4;CCL5;PRL;CD40LG;VEGFA;BMP4;ERBB2;IL20;CSF2;CSF1; TNFRSF11B;FIGF;CXCL11;IFNG;C5;IL1RN;PF4;CCL4;IL12A;IL7;TGFB2;TRIP6;CXCL5;CCL27;IL5;IL19; INHA;SPRED1;GDF15;CCL21;PIK3R1;OSM;CXCL10;MUC4;MIF;INHBA
Receptor Binding					
109	280	48	151	2.5x10^-14^	CSF3;HBEGF;IL8;EFNB3;BID;IRS1;TNF;CXCL12;CCL20;EREG;IL4;CCL5;LCK;NCK1;DLL4;ANXA1; NCOR2;PRL;NMB;CD40LG;TGFA;FBLN5;SLC9A3R1;VEGFA;ICAM2;GRN;NCOA6;EFNB1;THY1; BMP4;STOML2;ERBB2;IL20;CSF2;ANG;CSF1;PCSK9;ALCAM;FGF1;TNFRSF11B;CYTL1;VWF;GAST; FIGF;F2R;ADAM23;ADAM9;CXCL11;IFNG;COL4A3;TGFB1;GFRA1;IGF1;C5;GRB2;NPY;MED12; PTHLH;MBL2;EDN1;IL1RN;GABARAP;AREG;ADAMTS13;STC2;DLL1;PF4;CCL4;EFNA5;TGFB3; IL12A;EGF;IL7;TGFB2;TRIP6;CXCL5;CCL27;FGF3;IL5;WNT5A;SOCS2;FADD;TGFB1I1;F2;IL19; NUP62;INHA;TNXB;JMJD1C;GABARAPL2;SPRED1;GDF15;INHBC;RLN1;CCL21;CCR2;CHGB;SOCS1;DST;PIK3R1;OSM;CXCL10;MUC4;TDGF1;CASP8AP2;MIF;JAG2;SHC1;INHBA
Hematopoietin/Interferon Cytokine Receptor Activity					
8	68	5	48	1.7x10^-12^	CSF3;IL4;PRL;CSF2;IFNG;IL7;IL5;OSM
Growth Factor Activity					
18	79	8	51	1.0x10^-10^	CSF3;IL4;TGFA;GRN;CSF2;CSF1;FGF1;IL1RN;AREG;EGF;IL7;TRIP6;FGF3;IL5;INHA;TDGF1;JAG2; INHBA
Extracellular Region					
140	293	58	145	1.3x10^-8^	THBS4;CSF3;HBEGF;COMP;IL8;DSPP;CCL20;MMP3;EREG;IL4;IL6;TFF3;PLA2G5;CTGF;IL5RA;CPN1;CD5L;DGCR6;ORM1;FBLN5;PNLIPRP2;MMP9;VEGFA;DCD;S100A7;COL18A1;LEP;TFRC;IL15; CCL14;MMP2;PSAP;ERBB2;IL20;SERPINA5;IL18;LGALS3BP;ANG;IL1A;GSN;MMP7;PCSK9;SOD1; CDA;PRG2;CDH13;TNFRSF11B;CYTL1;CP;SFRP4;VWF;COL8A1;LGALS7;TPT1;SERPINA1;FIGF; CFHR1;SPINT2;FBN1;COL4A3;TGFB1;PI3;IL16;LOXL1;KLK5;C5;FGG;WNT2B;NPY;PTHLH;MBL2; EDN1;IL1RN;AREG;ADAMTS13;SFRP1;SFN;PRSS8;MMP13;CCL4;EFNA5;IL12A;TINAG;IL7;TGFB2; SHH;LAMC1;IGFBP1;FGF3;FRZB;MMP10;IL5;WNT5A;DKK3;KLK10;APCS;F7;MMP11;FLT1;APOD; LAMB1;ANGPTL4;CRLF1;CPB2;FBLN2;POSTN;PDZD2;LYZ;KLK6;MUC5AC;NUCB1;F2;C2;MSMB; INHA;FSTL1;TNXB;F13A1;PTX3;GDF15;KLK8;VTN;INHBC;KLK13;COL5A1;COL5A3;PLG;DST;OSM; FGF2;RBP4;ADM;A1BG;FGFBP1;LALBA;REG3A;FBN2;MIF;DMBT1;INHBA
Chemokine Activity					
12	70	9	49	1.7x10^-8^	IL8;CXCL12;CCL20;CCL5;CXCL11;C5;PF4;CCL4;CXCL5;CCL27;CCL21;CXCL10
Extracellular Space					
83	196	37	105	2.2x10^-8^	CSF3;HBEGF;IL8;CCL20;MMP3;EREG;IL4;IL6;IL5RA;CPN1;CD5L;ORM1;MMP9;VEGFA;LEP;IL15; CCL14;MMP2;PSAP;LGALS3BP;ANG;IL1A;MMP7;PCSK9;SOD1;CDH13;CYTL1;CP;SFRP4;LGALS7; TPT1;SERPINA1;FIGF;CFHR1;FBN1;TGFB1;IL16;KLK5;C5;FGG;WNT2B;NPY;PTHLH;MBL2;EDN1; IL1RN;AREG;SFRP1;SFN;PRSS8;MMP13;CCL4;EFNA5;IL12A;SHH;IGFBP1;MMP10;IL5;WNT5A; DKK3;APCS;FLT1;APOD;CRLF1;CPB2;LYZ;NUCB1;F2;C2;MSMB;INHA;FSTL1;KLK8;VTN;PLG;OSM; FGF2;RBP4;ADM;FGFBP1;LALBA;REG3A;INHBA
G-Protein-Coupled Receptor Binding					
14	75	10	51	2.3x10^-8^	IL8;CXCL12;CCL20;CCL5;SLC9A3R1;CXCL11;C5;PF4;CCL4;CXCL5;CCL27;CCL21;CCR2;CXCL10
Chemokine Receptor Binding					
13	72	9	49	2.4x10^-7^	IL8;CXCL12;CCL20;CCL5;CXCL11;C5;PF4;CCL4;CXCL5;CCL27;CCL21;CCR2;CXCL10
Extracellular Region					
106	227	46	118	7.3x10^-8^	THBS4;CSF3;HBEGF;COMP;IL8;DSPP;CCL20;MMP3;EREG;IL4;IL6;CTGF;IL5RA;CPN1;CD5L;DGCR6;ORM1;FBLN5;MMP9;VEGFA;COL18A1;LEP;IL15;CCL14;MMP2;PSAP;LGALS3BP;ANG;IL1A;MMP7;PCSK9;SOD1;CDH13;CYTL1;CP;SFRP4;COL8A1;LGALS7;TPT1;SERPINA1;FIGF;CFHR1;FBN1; COL4A3;TGFB1;PI3;IL16;KLK5;C5;FGG;WNT2B;NPY;PTHLH;MBL2;EDN1;IL1RN;AREG;ADAMTS13;SFRP1;SFN;PRSS8;MMP13;CCL4;EFNA5;IL12A;TINAG;SHH;LAMC1;IGFBP1;MMP10;IL5;WNT5A; DKK3;APCS;MMP11;FLT1;APOD;LAMB1;CRLF1;CPB2;FBLN2;POSTN;LYZ;MUC5AC;NUCB1;F2;C2;MSMB;INHA;FSTL1;TNXB;KLK8;VTN;COL5A1;COL5A3;PLG;DST;OSM;FGF2;RBP4;ADM;FGFBP1; LALBA;REG3A;FBN2;INHBA
Locomotory Behavior					
33	112	17	64	2.4x10^-7^	IL8;CXCL12;CCL20;IL4;IL10;CCL5;PIK3CB;SOD1;MAPK1;FOSL1;CDH13;PLD1;CXCL11;C5;CXCR4; DEFA1;PF4;CCR5;TGFB2;CXCL5;CCL27;MAP2K1;CCR7;CXCR3;PLAUR;RALBP1;CCL21;CCR2;CCR6; FGF2;HRAS;CXCL10;MAPK14
Membrane Fraction					
42	113	17	59	2.5x10^-7^	CEACAM1;VCP;BID;IRS1;EEA1;GALK1;LYVE1;IL15;BCAR1;FOLR3;FUT3;LY6D;ATP8B1;STX16;JUP; FUT4;STEAP2;CD59;HLAB;GP1BA;PRKCE;DPP10;APOB;XRCC6;SPTAN1;ABCB1;DAG1;CLIC1;CDH5;PTGS1;ABCA3;SLC1A1;LRP12;AMFR;PRKCA;SELP;PRKCZ;CD70;LRP1;PDLIM5;FOLH1;SLC2A1
Response to External Stimulus					
108	249	47	122	2.9x10^-7^	IL13;IL8;ITGB3;CXCL12;WAS;CCL20;EREG;IL4;IL10;CCL5;FOS;CD97;F13B;MMRN1;PLAT;ANXA1; CTGF;CD40LG;ORM1;RPS6KA5;RAC1;S100A8;LEP;LYVE1;SSTR2;F5;PIK3CB;MDK;SELE;IL20;TFPI; IL1A;IL1RAP;IL18RAP;PCSK9;SOD1;MAPK1;FOSL1;CDH13;S100A9;CD59;VWF;IL10RB;PLD1; CFHR1;F2R;GP1BA;GCGR;CXCL11;TGFB1;TP53;C5;CHST2;NMI;CXCR4;NPY;MBL2;DEFA1;STC2; PF4;CCR5;MGLL;CCL4;CRP;CDKN1A;CHMP1A;TNFAIP6;TGFB2;CXCL5;CCL27;NF1;IL5;APCS;F7; MAP2K1;CCR7;RTN4RL2;LYZ;CXCR3;PPARG;CDKN2B;F2;S100A12;AOAH;C2;SERPINE1;INHA; F13A1;PLAUR;PTX3;KLK8;RALBP1;PRDX5;CCL21;PLG;CCR2;CCR6;FGF2;HRAS;RIPK2;TACR1;ADM;CXCL10;PROC;MAPK14;ITGA2;RELA;INHBA
Regulation of Actin Filament Length					
4	53	6	35	8.1x10^-7^	CXCL12;NCK1;GSN;CAPG
Regulation of Actin Polymerization and/or Depolymerization					
4	53	6	35	8.1x10^-7^	CXCL12;NCK1;GSN;CAPG
Regulation of Cellular Component Size					
4	53	6	35	8.1x10^-7^	CXCL12;NCK1;GSN;CAPG
Behavior					
41	126	22	69	1.4x10^-6^	IL8;CXCL12;CCL20;IL4;IL10;CCL5;IL1RAPL1;CRHBP;LEP;PIK3CB;HPRT1;SOD1;MAPK1;FOSL1; CDH13;PLD1;CXCL11;C5;CXCR4;NPY;DEFA1;PF4;CCR5;TGFB2;CXCL5;CCL27;NF1;MAP2K1;CCR7; CXCR3;PLAUR;KLK8;RALBP1;CCL21;CCR2;CCR6;FGF2;HRAS;TACR1;CXCL10;MAPK14
Actin Polymerization and/or Depolymerization					
9	62	8	38	3.1x10^-6^	CXCL12;NCK1;RAC1;DSTN;ANG;GSN;WIPF1;CAPG;WASL
Regulation of Organelle Organization and Biogenesis					
11	65	9	39	5.2x10^-6^	CXCL12;KATNB1;NCK1;MAPRE1;GSN;ARAP1;APC;CAPG;NEXN;NF2;TSC1
Regulation of Cytoskeleton Organization and Biogenesis					
11	65	9	39	5.2x10^-6^	CXCL12;KATNB1;NCK1;MAPRE1;GSN;ARAP1;APC;CAPG;NEXN;NF2;TSC1
Response to Biotic Stimulus					
26	99	16	52	5.2x10^-6^	VCP;TNF;CXCL12;GSK3B;FGR;IL10;CCL5;S100A7;BCL3;HSPB1;CD24;IFNGR1;FOSL1;BCL2;PTPRC; TP53;CXCR4;TLR3;CCL4;IFNAR1;IL12A;VAPB;S100A12;AMFR;LALBA;DMBT1
Nucleus					
198	318	113	80	1.3x10^-5^	SUZ12;GADD45A;BCAS2;VCP;EGFR;HLF;AXIN2;IRS1;GSK3B;FOS;EGR1;KPNA2;BRCA1;CNOT7; SMAD4;CEP290;XPA;MDM2;PARK7;NBN;NCOR2;BRCA2;STK17A;LGALS3;DEK;FOXO3;RPS6KA5; HEXIM1;GZMB;MECP2;CDK1;S100A7;ERG;SUFU;PARP1;SIK1;BCL3;NCOA6;PTGS2;RAD54L; MCM2;PPIA;SPDYA;BIRC3;KDM5B;PTMS;DLGAP5;BCL6;PHB;TP63;CDK6;CRK;MYC;STIP1;NDC80;S100A11;ANG;PTGES3;SOD1;ABL1;BTG1;ACTB;USP4;PPARA;PPIE;FOSL1;FANCA;CYCS;BCL2; METTL3;CHEK1;STAT1;SIN3A;RALY;USF1;CCNA2;MGMT;ASH2L;WRN;GLI3;MALT1;ERCC5;EPC1; CDK9;ESR2;LYN;NFKB2;FOXO1;CDKN1B;TP53;HNRNPU;HTATIP2;APC;PIN1;MED12;PBX3;TBP; G3BP1;GNL3;CDK2;ATXN3;RXRB;FLNA;NOTCH4;MEF2A;CDKN2A;SRF;IGFBP3;USP7;NME1; GATA2;CCNB1;CREB1;MYCN;CHMP1A;CSNK2A1;EZH2;MAPKAPK3;PPM1D;JUN;RARB;NFKBIA; RGS12;XRCC6;PHB2;NPM1;MAML2;HIF1A;NF1;TLE1;TAF1;SMARCA2;SUMO1;SENP2;NAP1L1; NOTCH1;NAP1L3;AXIN1;CFL1;XRCC4;PDZD2;STAT3;CDKN2B;KDM5A;MSH6;CSTF1;CIDEA;CEBPG;PPIG;CDK4;NUP62;CLIC1;MSMB;EED;MRPL40;MLH3;PTGS1;MUTYH;KHDRBS3;LIMK2;NLRP5; RXRA;PA2G4;NOTCH2;DUSP4;ATF4;HDAC1;UBR5;RB1;CDKN2C;RAD50;RAD51;REC8;NF2;SENP1;RANBP1;HDAC2;PSEN2;CDK2AP1;VHL;NCBP2;UTP20;CCS;LSM1;ZBTB22;IRF9;BUB1B;EHD2;EIF6;RAD52;TOB2;ETS2;XRCC5;MAPK14;CASP8AP2;RELA;TRAF4;PTMA
Microtubule-Based Process					
13	67	11	40	2.3x10^-5^	CXCL12;KATNB1;NCK1;KPNA2;MAPRE1;GSN;ARAP1;TTK;APC;MAP7;CAPG;NF2;TSC1
Response to Other Organism					
20	87	14	46	3.5x10^-5^	TNF;CXCL12;FGR;IL10;CCL5;S100A7;BCL3;CD24;IFNGR1;FOSL1;BCL2;PTPRC;CXCR4;TLR3;CCL4; IFNAR1;IL12A;S100A12;LALBA;DMBT1
Cell Fraction					
76	181	38	82	3.8x10^-5^	CEACAM1;IL13;VCP;BID;IRS1;TNF;CD55;MDM2;NMB;CD40LG;FBLN5;CRHBP;EEA1;GALK1;LYVE1;AGT;FSHB;EFNB1;IL15;BCAR1;FOLR3;FUT3;LY6D;ATP8B1;STX16;DUSP6;JUP;FUT4;STEAP2;CD59;CYTL1;NTS;HLAB;SPINT2;GP1BA;ANXA2;TP53;PRKCE;WISP2;EDN1;DPP10;APOB;XRCC6; TNFSF13B;WNT5A;SPTAN1;CTTN;ABCB1;DAG1;F2;ACP1;S100A12;CLIC1;FAS;CDH5;PTGS1; ABCA3;SLC1A1;GCLM;LRP12;AMFR;PRKCA;UBR5;CCR2;SOD3;SELP;PRKCZ;CCS;ADM;CD70; WISP1;LRP1;REG3A;PDLIM5;FOLH1;SLC2A1

^1^ Among the total number of genes in the pathway, number of unique genes in our array data

^2^ Total number of corresponding antibody probes in the assay

^3^ Among the significant probes, via paired t-test comparing G&C to placebo, the number that have an effect size >0

^4^ Among the significant probes, via paired t-test comparing G&C to placebo, the number that have an effect size <0

^5^ GO = Gene Ontology; KEGG = Kyoto Encyclopedia of Genes and Genomes

*All pathways listed were statistically significant with Bonferroni correction (*P* <3.85x10^-4^ for KEGG pathway; *P*<5.18x10^-5^ for GO pathway)

Of the individual proteins, 508 were statistically significantly different between the two interventions with a Bonferroni correction (p < 1.7 x 10^-5^). The 100 most significant antibodies (p values 3 x 10^-16^ to 1 x 10^-9^) are given in Table 4.

**Table 4:** Top 100 (of 508) individual protein antibodies significantly different after glucosamine and chondroitin supplementation versus placebo intervention periods (n = 18).

Gene^1^	Function^2^	Effect size^3^	*P* value*
CEACAM1	Cell-cell adhesion	-2.45	8.7x10^-15^
SUZ12	Proliferation and histone methyltransferase activity [50,51]	1.09	1.7x10^-14^
THBS4	Cell-to-cell and cell-to-matrix interactions, extracellular mitogen	-1.88	2.1x10^-14^
GADD45A	Induced in response to DNA damage	-1.37	2.8x10^-14^
ITGA5	Adhesion and cell-surface mediated signaling	-1.54	9.7x10^-14^
ITGB4	Adhesion and cell-surface mediated signaling	-1.89	1.2 x10^-13^
CSF3(GCSF)^§^	Cytokine involved in hematopoiesis and induction of granulocytes	-3.06	2.1 x10^-13^
PKNOX1	RNA polymerase II distal enhancer	1.43	2.8 x10^-13^
IL13^§^	Immunoregulatory cytokine involved in inhibition of allergic reaction, particularly in the airways	-6.29	3.5 x10^-13^
C1orf38	Mediates macrophage inflammatory response	3.67	4.6 x10^-13^
SON	Splicing co-factor for cell-cycle progression and DNA-repair, involved in differentiation of hematopoietic cells	1.02	6.4 x10^-13^
MUC3B	Provides protective barrier against infectious agents at mucosal surfaces	3.83	1.3 x10^-12^
RUNX1	Subunit of transcription factor that binds to many enhancers and promoters, involved in development of normal hematopoiesis	3.93	1.4 x10^-12^
IL17D	Cytokine involved in the stimulation of other cytokines, e.g., IL6, IL8, and CSF	-2.27	1.6 x10^-12^
BCAS2	Component of pre-mRNA splicesome complex	1.72	2.3 x10^-12^
KCNE3	Modulates gating kinetics of potassium voltage channel complexes	1.75	3.2 x10^-12^
CD44	Cell adhesion and migration, receptor for hyaluronic acid	1.50	3.3 x10^-12^
VEPH1	Function unknown	1.80	3.7 x10^-12^
HBEGF	Normal heart function, smooth muscle cell proliferation, may be involved in macrophage mediated proliferation	-1.47	5.2 x10^-12^
VCP	Putative ATP-binding protein in vesicle transport and fusion, 26S proteasome function and assembly of peroxisomes	-2.10	6.8 x10^-12^
COMP	Structural integrity of cartilage, potent suppressor of apoptosis in chondrocytes	-2.08	7.4 x10^-12^
IL8^§^	Chemokine, chemoattractant and potent angiogenic factor	-2.35	9.9 x10^-12^
CAPN3 (NCL1)	Intracellular protease, binds to titin	-2.09	1.0 x10^-11^
GCM2	Transcription factor regulating parathyroid development	1.23	1.0 x10^-11^
PKC	Regulation of cell growth and immune responses	-0.89	1.3 x10^-11^
LASP1	Regulation of actin-based cytoskeletal activities	-1.42	1.4 x10^-11^
SPP1 (Osteopontin)	Attachment of osteoclasts to the mineralized bone matrix; also a cytokine that upregulates expression of interferon-gamma and interleukin-12	-6.45	1.7 x10^-11^
EFNB3	Ligand for Eph receptors involved in migration, repulsion and adhesion during neuronal, vascular and epithelial development	-3.24	1.9 x10^-11^
HOXA4	Transcription factor that may regulate gene expression, morphogenesis and differentiation	1.88	2.3 x10^-11^
IL1β	Cytokine involved in inflammatory response	-2.65	2.3 x10^-11^
EGFR^§^	Cell proliferation	1.80	3.1 x10^-11^
PRKCQ	Kinase involved in diverse cellular signaling pathways including T-cell activation, proliferation, differentiation and survival	-1.68	3.1 x10^-11^
ENO1	Multifunctional glycolytic enzyme involved in glycolysis, growth control, hypoxia tolerance and allergic response	-3.37	3.2 x10^-11^
SULF1	Inhibits signaling by heparin-dependent growth factors, diminishes proliferation and facilitates apoptosis	2.39	3.2 x10^-11^
MUC6	Modulates the composition of the protective mucus layer related to acid secretion or presence of bacteria in the lumen	1.43	3.4 x10^-11^
HDA-1	Histone deacetylase; regulation of gene expression	1.35	4.3 x10^-11^
TACSTD2	Cell surface receptor that transduces calcium signals	1.20	4.9 x10^-11^
AXIN2	Inhibitor of Wnt signaling pathway, down-regulates beta-catenin	1.89	5.1 x10^-11^
IRS1	Mediates the control of various cellular processes by insulin	-1.37	5.1 x10^-11^
VPS25	Formation and sorting of endosomal proteins destined for lysosomal degradation	-1.26	5.6 x10^-11^
ANKRD11	Inhibits ligand-dependent activation of transcription	1.34	5.8 x10^-11^
DEFA1;A1B	Antibacterial, fungicidal and antiviral activities	-1.01	6.5 x10^-11^
RASGRF2	Signal coordination of mitogen-activated protein kinase pathways	1.07	8.3 x10^-11^
ESM1	Expressed in endothelial cells in lung and kidney, regulated by cytokines	3.77	8.5 x10^-11^
DSPP	Dentinogenesis	2.18	8.6 x10^-11^
BAG1	Anti-apoptotic factor	1.52	9.3 x10^-11^
NOV	Cell-adhesion, migration, proliferation, differentiation and survival	-1.79	9.3 x10^-11^
LIFR^§^	Cytokine	-4.54	1.2 x10^-10^
EXT2	Heparin sulfate bioysynthesis	1.16	1.2 x10^-10^
CXCL12(SDF1)^§^	Ligand for G-protein coupled receptor involved in inflammation	-1.57	1.2 x10^-10^
CD55/DAF	Regulation of the complement cascade	1.90	1.3 x10^-10^
LEF1	Involved in Wnt signaling and enhances T-cell receptor binding	2.58	1.4 x10^-10^
LIN28B	Suppressor of microRNA biogenesis	1.40	1.4 x10^-10^
PRLR^§^	Cytokine receptor, interacts with prolactin	2.39	1.6 x10^-10^
MASP1	Complement activation	-1.99	1.7 x10^-10^
KRT10	Cytoskeleton composition	-1.56	1.8 x10^-10^
WAS	Signal transducer for actin cytoskeleton	3.76	1.8 x10^-10^
FSCN1	Actin-bundling protein	-1.48	2.0 x10^-10^
NAGLU	Degrades heparin sulfate	-1.85	2.1 x10^-10^
CCL20^x^	Chemotactic factor	4.00	2.5 x10^-10^
MMP3	Extracellular matrix degradation	1.61	2.6 x10^-10^
DLAT	Pyruvate dehydrogenase complex component which catalyzes the conversion of pyruvate to acetyl CoA	-1.88	3.0 x10^-10^
SP1	Activator or repressor of transcription of a battery of genes related to cell growth, apoptosis, differentiation and immune response	-1.46	3.0 x10^-10^
EREG	Epidermal growth factor family member	-4.04	3.0 x10^-10^
NOLA2	Ribosome biogenesis and telomere maintenance	3.92	3.1 x10^-10^
CALR3	Expressed in testis, may be required for sperm fertility	-1.22	3.2 x10^-10^
IL4^§^	Cytokine produced by activated T cells	-1.04	3.3 x10^-10^
ARHGEF17	Exchange factor for RhoA GTPases	1.86	3.3 x10^-10^
STMN1 (S15)	Regulation of filament system destabilization	3.34	3.3 x10^-10^
SPARC (Osteonectin)	Calcification of collagen in bone; also regulates cell shape and growth through interactions with extracellular matrix and cytokines	-1.05	3.3 x10^-10^
FGR	Negative regulator of cell migration and adhesion	1.60	3.5 x10^-10^
IL6^§^	Cytokine with diverse functions in inflammation, maturation of B cells, osteoblast formation, neuronal differentiation, hematopoiesis and energy mobilization in muscle	1.33	3.8 x10^-10^
CCL5^§^	Chemoattractant for monocytes, memory T helper cells and eosinophils	-2.78	4.4 x10^-10^
CSRP1	Cellular differentiation	-1.11	4.9 x10^-10^
PEPD	Recycling of proline, may be rate limiting for production of collagen	-1.27	5.0 x10^-10^
LCK	Key signaling molecule in selection and maturation of developing T-cells	1.22	5.2 x10^-10^
WNT3A	Cell-cell signaling during embyogenesis	-2.95	5.2 x10^-10^
DEPDC1	Transcriptional co-repressor	2.52	5.5 x10^-10^
KRT18	Uptake of thrombin-antithrombin complexes by hepatic cells; filament reorganization	-1.56	5.5 x10^-10^
UBC	Protein degradation	-2.00	5.6 x10^-10^
SERPINB5	Tumor suppressor	1.27	6.1 x10^-10^
CCSP-2	Marker for colon cancer	-1.69	6.1 x10^-10^
EGR1	Activates transcription of genes required for mitogenesis and differentiation	1.36	6.3 x10^-10^
DCN	Matrix assembly	1.85	6.4 x10^-10^
TNFRSF9^§^	TNF receptor family	-3.59	6.9 x10^-10^
CD142(F3)	Initiates blood coagulation	-1.29	7.3 x10^-10^
NPEPL1	Aminopeptidase activity	2.01	7.4 x10^-10^
KPNA2	Nuclear transport of proteins	2.01	7.7 x10^-10^
BRCA1	Tumor suppressor	2.08	7.7 x10^-10^
IER3	Anti-apoptotic factor	2.56	7.8 x10^-10^
PHLDA2	Tumor Suppressor	-2.73	8.2 x10^-10^
AK1	Cellular energy homeostasis and adenine nucleotide metabolism	2.40	8.5 x10^-10^
F13B	Coagulation factor	-3.38	8.5 x10^-10^
GJA1 (pS373)	Gap junction protein	1.99	8.8 x10^-10^
STX11	Protein transport	1.22	9.3 x10^-10^
TLN1/TLN2	Cytoskeletal protein	-1.64	9.3 x10^-10^
PPP2CA	Negative control of cell-growth and division	-2.08	9.4 x10^-10^
FUT8	Catalyzes addition of fucose in alpha 1–6 linkages	2.36	1.0 x10^-09^
ITGA1	Adhesion and cell-surface mediated signaling	-1.94	1.0 x10^-09^
CNOT7	Negative regulator of cell proliferation	-1.34	1.1 x10^-09^

^1^Indicates that protein was in the GO (Gene Ontology) or KEGG (Kyoto Encyclopedia of Genes and Genomes) Cytokines pathway

^2^Information pertaining to function is derived from PubMed Gene and/or UniProtKB unless otherwise noted

^3^Values >1 indicate greater antibody expression after supplementation with G&C compared to placebo; values <1 indicate lower expression

*All proteins listed were statistically significant with Bonferroni correction (*P* <1.70x10^-5^)

## Discussion

In this randomized, crossover trial, we found that G&C supplementation statistically significantly lowered mean serum CRP concentrations by 23% compared to placebo. In support of this finding, “Cytokine activity” was the most significant pathway altered between the two interventions in both the GO and KEGG gene-set enrichment analyses, and was lower after G&C. Correspondingly, a number of individual cytokines and other inflammation-related factors were significantly lower in the proteomics panel, including several interleukins and chemokines.

Our results are in agreement with a growing body of *in vitro* and animal research which suggests that G&C have anti-inflammatory properties. Laboratory studies indicate that G&C inhibit NF-κB, by preventing the degradation of its inhibitory subunit, Iκ-B [10]. Thus, NF-κB is unable to translocate to the nucleus, where this transcription factor activates the expression of a battery of genes involved in inflammatory response and cell proliferation [32]. These anti-inflammatory effects have been corroborated *in vivo* in animal studies. In rabbits with induced atherosclerosis and chronic arthritis, treatment with G inhibited NF-κB activation and down-regulated COX-2 expression in peripheral blood mononuclear cells, while also decreasing circulating levels of CRP and IL-6 [33]. Further research has shown that mice fed a G-containing diet for 56 days had lower serum IL-6 and TNFα than mice fed a control diet [34]. Two recent studies reported that G&C administration has anti-inflammatory effects in the colon [35]. Bak, et al [13], reported reduced TNFα, interleukin-1β, and NF-κB mRNA expression in colonic mucosa in mice induced with colitis after supplementation of G at 0.10% diet (wet weight) compared to control for four weeks. Finally, in rats with chemically-induced colitis, treatment with G ameliorated symptoms of colitis, while decreasing both systemic and colonic inflammation, as measured by serum concentrations of IL-8 and amyloid P component, and colonic expression of NF-kB, IL-1β, and TNFα, respectively [35]. These studies are notable given our findings of decreased colorectal cancer incidence with use of G&C in the VITAL study [4], and significantly decreased concentrations of CEACAM1 (carcinoembryonic antigen-related cell adhesion molecule 1) in the present study. CEACAM1 is an immunoglobulin involved in cell-to-cell adhesion, and its overexpression is implicated in colorectal cancer [36]. Further, this protein was the most significantly altered protein between the two interventions (effect size-2.45; *P* = 8.7x10^-15^).

Despite the compelling results from *in vitro* and preclinical models, few studies have evaluated the effects of G&C on inflammation in humans. In a nationally representative sample of nearly 10,000 adults included the National Health and Nutrition Examination Study (NHANES), regular use of G&C compared to nonuse was associated with a ~20% lower mean concentrations of hsCRP [14]. In addition, we recently observed G&C use to be associated with 28–36% lower CRP concentrations, [37] and 40–47% lower oxidative stress, as measured by urinary prostaglandin F_2_α (PGF2α) [38] among a sub-set of 220 individuals in the VITAL cohort [15]. Only two randomized trials of G &/or C on markers of inflammation have been conducted. In a small trial, 36 osteoarthritis patients were given 1500 mg G and 675 mg C for twelve weeks [39]. A significant reduction in serum PGE_2_ was observed among the G&C treated group, with post-treatment levels similar to that of 25 age-matched healthy controls. The same group conducted a second trial in which 51 rheumatoid arthritis patients were randomized to receive either 1500 mg G or placebo for twelve weeks [40]. While treatment with G reduced serum MMP-3 levels, CRP concentrations remained unchanged. However, exposure was limited to G alone (without C) and patients continued taking regular medications throughout the study. Furthermore, as individuals with rheumatoid arthritis have higher levels of inflammation than the general population, the results of this study may not be generalizable to a healthy population.

Additional inflammation-related pathways that were significantly different between interventions with Bonferroni correction included JAK/STAT signaling, a signaling pathway for a broad range of cytokines and growth factors [41]; intestinal immune network for IgA production, which serves as the first line of defense against microorganisms in the intestinal mucosa [42]; leukocyte transendothelial migration, the movement of leukocytes from blood into tissues for immune surveillance and inflammation [43]; and hematopoietin/interferon cytokine receptor binding, which includes class I cytokines, mainly the interleukins, and growth factors. G-protein coupled receptors are involved in diverse biologic functions, but include roles in growth and regulation of inflammation [44]. As with the cytokine pathway, the majority of the probes in these pathways were less abundant after G&C as compared to placebo. In fact, of the 30 significant pathways, the majority of probes were less abundant in all but two pathways—ubiquitin-mediated proteolysis and proteins contained in the nucleus. Alterations in these other inflammation-related pathways suggest that G&C may have wider-ranging effects on inflammation beyond inhibition of transcription factor NF-kB.

Significant differences in other pathways between the interventions indicate that G&C may play a role in other biologic functions that have not been previously associated with this supplement. For example, pathways related to microtubule function, regulation of actin polymerization, filament length, cytoskeleton organization, and locomotory activity were lower after G&C. Although there is overlap among some genes representing the pathways, these differences suggest that G&C may have effects on cell division and motility, targets of a growing number of chemotherapeutic drugs [45–47]. Lower protein concentrations in pathways related to external and biotic stimulus, and other organisms were likely related to reduction in inflammation-related signaling, particularly given the antibodies contained on our assay. Other pathways that differed between the interventions were based on cellular component or location. The meaning of these differences in unrelated proteins, in terms of health consequences, is unclear.

Among the most significant individual proteins that were differentially abundant between the interventions in the proteomics analysis, several in the top ten were related to cell-adhesion (CEACAM, THBS4, ITGA and B). Other proteins with large effect sizes were IL-13 (down; -6.29), an immunoregulatory cytokine involved in inhibition of allergic reaction, particularly in the airways; osteopontin (down; -6.45), which is involved in the attachment of osteoclasts to the mineralized bone matrix, and is also a cytokine that upregulates the expression of interferon-gamma and IL-12; and C1orf38 (up; 3.67), involved in mediating macrophage inflammatory response. Consistent with the lower CRP concentrations measured in serum, CRP was also down-regulated in plasma on the antibody array (down; -0.5, P = 0.001) but was not significant with correction for multiple testing. As has been observed in many other serum/plasma studies [48,49], some of the proteins with significant changes are normally cytoplasmic or nuclear, so their role as putative serum/plasma biomarkers is unclear. They could indicate sufficient apoptosis and/or necrosis of cells to be detectable and imply that this is reduced with G&C treatment.

This is the first randomized trial to evaluate the effects of G&C on inflammation in healthy adults, and the first to assess potential pathways perturbed by G&C using a broad proteomic screen and gene set enrichment analyses. A major strength of this study is that participants were healthy and free of underlying inflammatory conditions, and the stringent inclusion and exclusion criteria minimized effects of other factors that may affect inflammatory status, (e.g., age, tobacco or medication use, chronic health conditions). Despite the small sample size, many of the intervention effects in the proteomics pathway analyses and for individual proteins were highly significant, exceeding by orders of magnitude, the Bonferroni corrections and the effects observed in multiple previous analyses we have performed in which we compared plasma samples in cancer cases and controls [23,26,27]. Detection of these highly significant effects appears to be due to our use of the randomized, crossover, placebo-controlled design where each participant acted as his or her own control. A strength of the platform is the significant coverage of the proteome including many cytokines (~3,000 proteins). Thus, it is a powerful approach for identifying pathways that change in response to an intervention. Furthermore, it is a powerful discovery method for identifying individual biomarkers that may play an important role in the process being studied. Individual protein results warrant validation by other means such as ELISA or immunoblot since the array uses only a single antibody to bind the protein from plasma and the indicated antibody could bind a protein in plasma that is not the one claimed by the manufacturer (i.e., the specificity of each antibody has not be determined). Finally, given the sample size of the current study, the anti-inflammatory properties of G&C need to be replicated in larger randomized trials and should evaluate sex-specific effects.

In summary, we found that G&C significantly reduced circulating CRP concentrations compared to placebo, and gene set enrichment analyses indicated that cytokine activity and other inflammation-related pathways were significantly decreased. These results are supported by *in vitro* and animal studies demonstrating anti-inflammatory properties of G&C, as well as human observational data which show an association between G&C use and lower concentrations of circulating CRP. Thus, there is now growing evidence that G&C reduce systemic inflammation in humans. This evidence may also provide a possible biologic mechanism to support prior findings that use of G&C supplements is associated with reduced lung and colon cancer and overall mortality. Future studies in larger samples and other populations are needed to determine the potential utility of G&C as a possible anti-inflammatory agent.

## Supporting Information

S1 CONSORT Checklist CONSORT 2010 checklist of information to include when reporting a randomized trial. (DOC)

S1 Data File Serum inflammation and oxidative stress markers. (XLSX)

S2 Data File Plasma proteomics. (XLS)

S1 Protocol Glance study protocol. (DOC)
