# Supplementary material for: Randomized Trial of Glucosamine and Chondroitin Supplementation on Inflammation and Oxidative Stress Biomarkers and Plasma Proteomics Profiles in Healthy Humans
Source: PLoS One. 2015 Feb 26;10(2):e0117534. doi: 10.1371/journal.pone.0117534 (PMC4342228; doi:10.1371/journal.pone.0117534)
Supplement: S1 Protocol — (DOC) [file pone.0117534.s004.doc]

# GLANCE PROTOCOL

# TABLE OF CONTENTS

**1.1. Protocol Synopsis**

**1.2. Background/significance**

**1.3. Objectives and Aims**

**1.4. Study Design**

**1.5. Study Population/Recruitment**

**1.6. Eligibility Criteria**

- 1. **Key Measurements and Sample Handling**
  2. **Laboratory Procedures**
  3. **Data Analysis Plans**
  4. **Timeline**
  5. **Data Safety and Monitoring Plan**

**1.1. PROTOCOL SYNOPSIS**

**A Pilot Trial of Glucosamine and Chondroitin Effects (GLANCE)**

Chronic inflammation is associated with carcinogenesis. Increased serum concentrations of C-reactive protein (CRP) have been associated with human cancer risk. Glucosamine and chondroitin (G&C) are among the most commonly used dietary supplements in the US. In vitro and limited animal studies show that G&C have anti-inflammatory properties, including inhibition of Nuclear Factor-kappa B (NF-kB), a transcription factor and central mediator in the immune response. In a large prospective study of the association of dietary supplement use and cancer risk, we found that use of G&C was associated with a 26-35% risk reduction of lung and colorectal cancer, and a 16-17% reduction in total mortality; the strongest and most consistent results across endpoints of a beneficial effect of any supplement studied so far. Our goal in the proposed study is to examine the effect of G&C on objective markers of inflammation in order to evaluate its potential as a chemopreventive agent. Metabolomics analyses will also be carried out in serum to determine other potential mechanisms of action of G&C.

**Design:** A randomized, placebo-controlled trial

**Sample Size:** Recruitment of 30 participants with the goal of having 20 finish

**Participants:** Men and women

**Selection:** Individuals from the Greater Seattle area who are overweight (BMI of

25-30 kg/m^2^) and between the ages of 20-55

**Exclusion:** Exclusion criteria address medical and practical issues: 1) chronic medical illness,

history of gastrointestinal, hepatic, or renal disorders, or inflammatory conditions

(including autoimmune and inflammatory diseases); 2) pregnancy or lactation; 3)

currently on a weight-loss diet; 4) BMI (body mass index) <25 or >30 kg/m^2^; 5) alcohol intake

of greater than 2 drinks/day (2 drinks being equivalent to 720 ml beer, 240 ml wine or

90 ml spirits); 6) current use of prescription or over-the-counter medications other than

multivitamin pills or infrequent use of aspirin and NSAIDs, including oral contraceptives

and hormone-secreting IUDs, or use of aspirin or NSAIDs more than 2 days per week;

7) abnormal renal, liver or metabolic test 8) inability to swallow pills; 9) known allergy to

shellfish; 10) not willing to take pills made from shellfish or animal sources; 11) intention

to relocate out of study area within next 4 months; In the event that we cannot recruit

sufficient participants within a BMI range of 25-30, we will expand eligibility to

5<BMI<32.5.

**Intervention:** a) Placebo and b) an over-the-counter glucosamine and chondroitin supplement

(1500 mg and 1200 mg, respectively)

**1.2. BACKGROUND/SIGNIFICANCE**

Use of dietary supplements and aspirin is routinely adopted by the US public to reduce risk of chronic diseases, including cancer. Chronic inflammation contributes to carcinogenesis, and biomarkers of inflammation [e.g., C-reactive protein (CRP)] have been associated with cancer risk. Use of aspirin, an anti-inflammatory drug, has have been shown to reduce colorectal and lung cancer risk in randomized controlled trials. However aspirin and other non-steroidal anti-inflammatory agents have not been recommended for cancer chemoprevention due to their associated adverse effects.

Glucosamine and chondroitin (G&C), often taken together in a single pill, are the most commonly used non-vitamin, non-mineral “specialty” supplements in the US. They are considered safe with no known major adverse effects. Laboratory studies show that G&C inhibit the transcription factor Nuclear Factor-kappa B (NF-kB), leading to reduced expression of inflammatory genes and inhibition of cell proliferation, effects associated with lower cancer risk. Several lines of evidence support a possible role for G&C in reduction of inflammation and cancer. In a large prospective cohort study over a 10-year period (n=77,738), we observed a 26-28% reduction in lung cancer incidence, a 27-35% reduction in colorectal cancer risk, and a 16-17% reduction in total mortality with use of G&C. These effect sizes are comparable to those reported for aspirin. In addition, in a nationally representative sample of 10,000 adults (NHANES), we found that regular use of G&C was associated with ~20% reduced levels of CRP, a well-recognized marker of inflammation (*Am J Epidemiol, in press*). Furthermore, increased CRP has been associated with cancer risk in several observational cohort studies.

Despite these intriguing results, and evidence for biologic mechanisms, to date there have been no human intervention trials evaluating the potential for G&C to reduce inflammation or alter other pathways relevant to carcinogenesis in humans. Our previous findings, representing the only evidence on G&C in humans, are observational and are thus subject to bias. A randomized controlled trial (RCT) would directly assess the effect of G&C on inflammation while maximizing internal validity. We propose to conduct a pilot RCT using a crossover design of 2 treatments—placebo and G&C — in a sample of 30 overweight participants, 20-45y. We will test the effect of G&C on the circulating inflammatory biomarker CRP and serum metabolomic patterns. Metabolomics measures a broad range of metabolites present in a biologic sample to permit the characterization of whole-system responses to an exposure, and will allow us to explore other potential pathways modulated by G&C.

**1.3. SPECIFIC AIMS:**

1. To determine the effect of a common starting dose of G&C [1500 mg/d glucosamine hydrochloride + 1200 mg/d chondroitin sulfate] on serum hsCRP concentrations.

2. To characterize the intervention-induced changes in serum metabolomic patterns via pathway analyses in response to G&C as compared to placebo.

We hypothesize that treatment with G&C will reduce serum hsCRP concentrations. We further hypothesize that the serum metabolomic patterns will differ between treatment groups, however, this aim is exploratory as there is very little known about the mechanisms of action of G&C. This analysis will be instructive in determining unknown pathways that might be affected by G&C.

**1.4. STUDY DESIGN**

The study is designed as a randomized, cross-over, placebo-controlled intervention of G&C and placebo. We will randomize participants to one of the two treatment orders, either G&C first followed by the placebo in the second period or the placebo first followed by G&C. Each treatment will be 28 days in duration with at least a 28 day washout period between treatments. Blood will be drawn once at baseline and at the end (Day 28) of each treatment period. In the case of illness, and during Days 1-3 of a women’s menstrual cycle, participants will delay the start or end of a treatment sequence and corresponding clinic visit in order to avoid blood draws during transitory spikes in the inflammatory biomarkers of interest.

**1.4.a. G&C Supplements**

The active treatment period will include 1500 mg/d glucosamine hydrochloride + 1200 mg/d chondroitin sulfate. The test agent and placebo will be provided by NutraMax, a well-established supplement company that follows Good Manufacturing Practices.

**1.4.b. Safety and Adverse Effects**

G&C are among the most commonly used non-vitamin, non-mineral over the counter supplement in the U.S. Though there are many short-term studies in humans evaluating its efficacy in treating osteoarthritis, few adverse events have been reported. The largest human trial, GAIT, conducted in men and women ≥40y, had 952 participants total in the three G &/or C arms, and a duration of 24 weeks. In that trial, 3.7% of participants withdrew due to adverse effects. Only two adverse events were attributed to G&C treatment: chest pain in a subject receiving G and congestive heart failure in a patient receiving combined treatment; but no rationale for the attributions to the treatment were given. Other side effects were mild, e.g., stomach upset, and were evenly spread across treatment groups, including placebo. Because G contains a sugar moiety, one concern had been that it may induce or worsen insulin resistance. However, a recent review of the human and animal studies concluded that G does not cause glucose intolerance nor does it affect glucose metabolism. In many countries, G&C are approved drugs based on their safety and efficacy. The European League Against Rheumatism (EULAR) rated G as having a safety profile of 5 on a 0-100 scale and C a 6, two of the safest drugs for osteoarthritis. There are no known adverse drug-drug interactions with G or C. Based on follow-up of the VITAL cohort study of 77,000 men and  women,  those who used glucosamine (with or without chondroitin ) at baseline were at  16% lower risk of death (HR= 0.82; 95% CI (0.75-0.90)), 12% lower risk of CVD death (HR=0.88 (0.74-1.06)) and 13%  lower  risk of death from  cancer  (HR = 0.87 (0.76-0.98)).

**1.4.c. Summary of Study Activities and Measures**

| **Baseline** | | **Reason for Measures** |
| --- | --- | --- |
|  | Screening Questionnaire Exclusion criteria  Information session & Informed Consent | |
|  | Health and Demographics Questionnaire | Data collection |
|  | Food Frequency Questionnaire (FFQ - FHCRC) | Ascertain dietary intake |
|  | Blood draw | Liver and kidney function panel |
|  | 12 h urine collections | Future measures (e.g., PGE)  Treatment compliance (G&C metabolites)  Completeness of urine collection (creatinine) |
|  | DXA scan | Ascertain body fat % |
| **Randomized, cross-over with 2 intervention periods: G&C and placebo** | | |
|  | Fasting blood draws (end of each period) | hsCRP concentrations;  Metabolomics analysis  Future measures (e.g., proteomics) |
|  | 12 h urine collections (end of each period) | Future measures (e.g., PGE-M, F2iso)  Treatment compliance (G&C metabolites)  Completeness of urine collection (creatinine) |
|  | Spot urine (women; beginning of each period) | Rule out pregnancy |
|  | Daily calendar, pill check-off | Compliance |

**1.5. STUDY POPULATION/RECRUITMENT**

Participants will be healthy, non-smoking men and women, ages 20-55 y, who are overweight [body mass index (BMI) ≥25 and ≤30 kg/m^2^]. We will recruit study volunteers by distributing flyers throughout the community and local institutions of higher education and creating a study website. All recruitment materials will provide a description of the study and contact information. Study information and an initial screening questionnaire will be available online, which can be submitted through electronic or ground mail. A contact telephone number and email address will also be provided for individuals who want to be mailed an information packet. The screening questionnaire will include questions on medical history, medication use, etc, that relate to eligibility. The prospective participant will be given information about the study including: attendance requirements, time commitments, as well as information about the intervention study. Interested persons will return the completed questionnaire to the study staff, who will determine eligibility and will contact eligible persons by phone or email to schedule a meeting. A packet containing a confirmation letter and the screening study consent form will be sent to the person prior to attending the meeting. Participants eligible to enter the intervention will meet with our staff to discuss all the details and expectations of the study, review informed consent and answer any questions they might have.

**1.6. ELIGIBILITY CRITERIA**

Inclusion: Healthy men and women aged 20-55 years who are overweight (BMI of 25-30 kg/m^2^)

Exclusion: 1) chronic medical illness, history of gastrointestinal, hepatic, or renal disorders, or

inflammatory conditions (including autoimmune and inflammatory diseases)

2) pregnancy or lactation

3) currently on a weight-loss diet

4) BMI (body mass index) <25 or >30

5) alcohol intake of greater than 2 drinks/day (2 drinks being equivalent to 720 ml beer,

240 ml wine or 90 ml spirits)

6) current use of prescription or over-the-counter medications, including oral

contraceptives and hormone-secreting IUDs, or use of aspirin or NSAIDs more than 2

days per week

7) abnormal renal, liver or metabolic test

8) inability to swallow pills

9) known allergy to shellfish

10) not willing to take pills made from shellfish or animal sources;

11) intention to relocate out of study area within next 4 months

**1.7. SPECIMEN AND DATA COLLECTION**

**17.a. Blood** We will obtain 12-hour fasting blood samples at baseline for clinical chemistries (to confirm eligibility) and at the end of each 28-day intervention period. Participants will come to the Prevention Center where certified personnel will collect four 10-ml vacutainers of blood; two containers will be used for the present study and two will be stored for future use, e.g., proteomics, with obtainment of additional funding. Specimens will be stored at -80ºC.

**17.b. Urine** Participants will collect a 12-hour urine at baseline and at the end of each intervention period. Volume will be measured and urine stored at -80ºC. These samples will be used for future measurement of prostaglandin E (PGE-M), a surrogate for COX-2 enzyme activity involved in the inflammatory cascade, and other future assays.

**17.c. Dietary Assessment** We will use an established, validated food frequency questionnaire (FFQ) to characterize food and nutrient intakes of the study participants over the 3 months prior to baseline.

**17.d. Body Fat (BF)%** Although we will recruit overweight individuals with a BMI between 25-30, there will still be a range of BF%. Thus we will use Dual-X-ray absorptometry (DXA) in the Prevention Center to accurately ascertain BF%.

**1.8. LABORATORY PROCEDURES**

**1.8.a. Urinary Creatinine** concentrations, measured on a COBAS Mira analyzer, will be used to adjust for variation in 12-h urinary collections. Urines will be used for PGE measurement when future funds are obtained.

**1.8.b. Serum hsCRP Assays** We will measure hsCRP using CRP Ultra Wide Range reagent (Genzyme, Framingham, MA) on a Roche Cobas Mira Plus Chemistry Analyzer. Samples will be run in duplicate with blinded QCs in each batch; any sample with duplicate coefficients of variation >10% will be re-analyzed. Any extreme values will be excluded, determined by values >5 standard deviations (SD) from the median and >2 SD from any other data point, in order to eliminate any potential acute inflammatory states at any time points.

**1.8.c.** **Serum Metabolomics Analysis** Assays will be performed by Metabolon ([www.Metabolon.com](http://www.Metabolon.com/)) using patented, proprietary methods for detection and quantification. The analysis utilized 3 analytical platforms, LC/MS/MS(+ESI), LC/MS/MS(-ESI) and GC/MS, to separate and detect a broad range of small molecules (LC-MS/MS spectra). Once ions of interest are identified, their chemical identities are matched against an in-house standardized library of >3,000 human metabolites.

**1.9. DATA ANALYSIS**

**1.9.a. Data Handling and Quality Assurance Procedures** All participants enrolled in the study will be issued a computer-generated study ID. This ID will be used on all participant questionnaires and forms, specimen tracking forms, and cryovials. This method maintains participant confidentiality and facilitates data management. The labels used on the cryovials for the blood and urine samples are freezer-proof. Study data are identified by ID number only. A password-protected file links the participant name with the study ID.

**1.9.b. Data Analysis** All statistical analyses will be conducted using STATA (v12) software. We will examine the distributions of the baseline characteristics and the primary outcomes of the study participants. We will characterize the distributions using scatter plots, histograms, and summary statistics (e.g., mean, median, standard deviation, quartiles, range) to identify extreme outliers and assess normality. If the test indicates that the distribution is not normal or is skewed, then we will perform appropriate transformations prior to our primary analyses. From our experience and relevant literature, the logarithmic transformation is appropriate for the inflammatory biomarkers.

**1.9.c. Testing of Specific and Secondary Aims**

**Specific Aim 1:**

We will use a generalized estimating equation (GEE) modification of linear regression. This method will allow us to account for within-person variation over time as well as the correlation between the intervention-induced changes within an individual. The dependent variable lnCRP will be denoted by Yit of ith participant at time t, where t is 0 or 1 for either the beginning or end of each treatment period. Let Xi be the indicator variable for the intervention period with G&C. The linear model takes the form:

E[Y_it_] = β_0_ + β_1_ t + β_2_ X_i_ + β_3_ X_i_ t ; Var[Y_ij_] = σ^2^, Cov[Y_ij_, Y_kl_] = σ^2^ρ if i = k and j ≠ l, and 0 otherwise.

The parameter β_3_ is our main interest for Specific Aim 1 which represents the mean difference in CRP change from the pre- to post-treatment between the G&C and the placebo periods. Adjustment for potential confounders may not be needed due to randomization; however, we will evaluate other covariates for the purpose of reducing any unexpected confounding effects. All statistical tests will be two-sided with α set at 5%.

**Specific Aim 2:**

Statistical differences in serum metabolites between experimental groups will be evaluated using paired t-tests and False Discovery Rates (q-values).

**Power and Sample Size Considerations.** Our study is designed to yield 20 participants who will

complete the study. Based on the mean and SD for CRP in the 1999-2000 NHANES data restricted to our inclusion criteria (20-55y and BMI 25-30) and an intra-class correlation coefficient for CRP ranging from 0.60-0.80, we estimate 80% power to detect a 40-56% change in CRP with the probability of a type I error set at 0.05. As with any pilot study our power is limited. However information from this pilot study will be used to support a future R01 submission for a larger RCT, including information on: methods of recruitment and compliance, estimated effect size from a randomized trial and sample size needed, and new G&C pathways that may serve as additional biomarker endpoints.

**1.10. Project Timeline**

Our intention is to complete this project in 12 months; however, we have allocated up to 2 years in the event that we have a delay in recruitment of desired study participant population. During the first 2 months of the study, IRB materials will be created, data entry systems will be organized; forms will be printed. Starting in the third month, 30 participants will be recruited on a rolling-enrollment basis for participation in the intervention, with the goal of 20 completed all study activities. Liver and kidney function panels will be conducted routinely to ensure that participants can be enrolled on an ongoing basis. Biological samples from the intervention will be stored until each participant has completed both treatments so that samples for each participant can be analyzed together to minimize assay variation. Data analysis will begin, and the manuscript will be prepared during months 11 and 12.

|  | **1** | **2** | **3** | **4** | **5** | **6** | **7** | **8** | **9** | **10** | **11** | **12** |
| --- | --- | --- | --- | --- | --- | --- | --- | --- | --- | --- | --- | --- |
| Start up, Forms, IRB |  |  |  |  |  |  |  |  |  |  |  |  |
| Recruitment |  |  |  |  |  |  |  |  |  |  |  |  |
| G&C Intervention |  |  |  |  |  |  |  |  |  |  |  |  |
| Data Analysis |  |  |  |  |  |  |  |  |  |  |  |  |
| Manuscript Preparation |  |  |  |  |  |  |  |  |  |  |  |  |

**1.11. DATA SAFETY AND MONITORING PLAN**

Oversight for this study will be provided by the Principal Investigator, Dr. Emily White, with delegation of responsibilities to the Project Director, Dr. Sandi Navarro. They will ensure all entry criteria are met prior to the initiation of the protocol, and all study procedures and reporting of adverse events are performed according to the IRB-approved protocol.

All adverse events related to the study procedures will be fully documented on the appropriate case report form(s) and entered in a study database. For each adverse event, the investigator will provide the onset, duration, intensity, treatment required, and outcome, including documentation of need for premature termination of any study procedures.

**Anticipated adverse events related to this study are:**

**Glucosamine and Chondroitin (G&C):** G&C are among the most commonly used supplements in the U.S. In large human intervention trials, only mild symptoms, e.g., stomach upset, have been reported. The potential for unexpected adverse experiences to G&C are minimal. Participants will keep a daily study log which will include questions on health status and possible adverse experiences. Study staff will contact study participants at least bi-weekly while they are in a treatment period (including placebo) by phone (or email if the participant so requests) to ask about possible adverse experiences, assess compliance and address any questions or problems the participant may be having. The study manager has an emergency cell phone and can be reached 24/7 by study participants. The phone number will appear prominently on all study materials including the daily diary.

**Blood Draws:** Participants may experience a little discomfort or have a temporary bruise from having blood drawn. Occasionally a participant may feel lightheaded or feel faint when having blood drawn. Blood will be taken only by trained personnel using established procedures. The participants will be sitting down when the blood is drawn. If a participant feels faint, they will be instructed to lie down until the feeling goes away.

Adverse event grading scale:

**0= No Adverse Event** or within normal limits

**1= Mild Severity:** Transient laboratory test alterations; discomforts noted but no disruption of daily activities; no therapy, or only symptomatic therapy required
**2= Moderate Severity:** Laboratory test alterations indicating injury without long-term risk; discomfort sufficient to modify normal daily activity; specific therapy required (i.e., more than symptomatic)
**3= Serious Severity:** Laboratory test indicating a serious health threat or permanent injury; incapacity, inability to work, inability to perform normal daily activity; hospitalization required or prolonged; emergency treatment required; life-threatening events; death

**Plan for unanticipated AE reporting:** All unanticipated AEs related to the study procedures that are severe or serious will be reported by Drs. Emily White or Sandi Navarro to the IRB within 7 days of notification of the investigator

**Plan for anticipated AE reporting**: All serious anticipated AEs related to the study procedures will be reported by Drs. Emily White or Sandi Navarro to the IRB within 7 days of notification of the investigator

**Plan for ongoing review of results:** The PI will be notified within 24 hours by the research manager of any early terminations due to an adverse event

**Plan for safety review:** the PI will perform a cumulative review of all adverse events and premature terminations review every 6 months after study initiation or after completion of 50% of participant visits, whichever occurs first

**Plan for annual reporting:** A summary of the investigation including all adverse events and how they were handled, enrollment, drop-outs and reason for discontinuation and any protocol modifications will be provided to the IRB on an annual basis

Final Report will contain:

1. The number of adverse events and an explanation of how each event was handled
2. The number of complaints and how each complaint was handled
3. The number of subject withdrawals and an explanation of why the subject withdrew or was withdrawn
4. The number of protocol deviations and how each was handled

**References cited**

1. Qato, D.M., et al., *Use of prescription and over-the-counter medications and dietary supplements among older adults in the United States.* JAMA, 2008. **300**(24): p. 2867-78.

2. Coussens, L.M. and Z. Werb, *Inflammation and cancer.* Nature, 2002. **420**(6917): p. 860-7.

3. Thun, M.J., S.J. Henley, and T. Gansler, *Inflammation and cancer: an epidemiological perspective.* Novartis Found Symp, 2004. **256**: p. 6-21; discussion 22-8, 49-52, 266-9.

4. Heikkila, K., et al., *Associations of circulating C-reactive protein and interleukin-6 with cancer risk: findings from two prospective cohorts and a meta-analysis.* Cancer Causes Control, 2009. **20**(1): p. 15-26.

5. Rothwell, P.M., et al., *Long-term effect of aspirin on colorectal cancer incidence and mortality: 20-year follow-up of five randomised trials.* Lancet, 2010. **376**(9754): p. 1741-50.

6. Rothwell, P.M., et al., *Effect of daily aspirin on long-term risk of death due to cancer: analysis of individual patient data from randomised trials.* Lancet, 2011. **377**(9759): p. 31-41.

7. Bonovas, S., et al., *Cancer chemoprevention: a summary of the current evidence.* Anticancer Res, 2008. **28**(3B): p. 1857-66.

8. Wolfe, M.M., D.R. Lichtenstein, and G. Singh, *Gastrointestinal toxicity of nonsteroidal antiinflammatory drugs.* N Engl J Med, 1999. **340**(24): p. 1888-99.

9. Solomon, S.D., et al., *Cardiovascular risk of celecoxib in 6 randomized placebo-controlled trials: the cross trial safety analysis.* Circulation, 2008. **117**(16): p. 2104-13.

10. Bruno, J.J. and J.J. Ellis, *Herbal use among US elderly: 2002 National Health Interview Survey.* Ann Pharmacother, 2005. **39**(4): p. 643-8.

11. Jordan, K.M., et al., *EULAR Recommendations 2003: an evidence based approach to the management of knee osteoarthritis: Report of a Task Force of the Standing Committee for International Clinical Studies Including Therapeutic Trials (ESCISIT).* Ann Rheum Dis, 2003. **62**(12): p. 1145-55.

12. Chan, P.S., J.P. Caron, and M.W. Orth, *Short-term gene expression changes in cartilage explants stimulated with interleukin beta plus glucosamine and chondroitin sulfate.* J Rheumatol, 2006. **33**(7): p. 1329-40.

13. Iovu, M., G. Dumais, and P. du Souich, *Anti-inflammatory activity of chondroitin sulfate.* Osteoarthritis Cartilage, 2008. **16 Suppl 3**: p. S14-8.

14. Largo, R., et al., *Glucosamine inhibits IL-1beta-induced NFkappaB activation in human osteoarthritic chondrocytes.* Osteoarthritis Cartilage, 2003. **11**(4): p. 290-8.

15. Surh, Y.J., *NF-kappa B and Nrf2 as potential chemopreventive targets of some anti-inflammatory and antioxidative phytonutrients with anti-inflammatory and antioxidative activities.* Asia Pac J Clin Nutr, 2008. **17 Suppl 1**: p. 269-72.

16. Satia, J.A., et al., *Associations of herbal and specialty supplements with lung and colorectal cancer risk in the VITamins and Lifestyle study.* Cancer Epidemiol Biomarkers Prev, 2009. **18**(5): p. 1419-28.

17. Pocobelli, G., et al., *Total mortality risk in relation to use of less-common dietary supplements.* Am J Clin Nutr, 2010. **91**(6): p. 1791-800.

18. Allin, K.H., S.E. Bojesen, and B.G. Nordestgaard, *Baseline C-reactive protein is associated with incident cancer and survival in patients with cancer.* J Clin Oncol, 2009. **27**(13): p. 2217-24.

19. Il'yasova, D., et al., *Circulating levels of inflammatory markers and cancer risk in the health aging and body composition cohort.* Cancer Epidemiol Biomarkers Prev, 2005. **14**(10): p. 2413-8.

20. Siemes, C., et al., *C-reactive protein levels, variation in the C-reactive protein gene, and cancer risk: the Rotterdam Study.* J Clin Oncol, 2006. **24**(33): p. 5216-22.

21. Trichopoulos, D., et al., *Plasma C-reactive protein and risk of cancer: a prospective study from Greece.* Cancer Epidemiol Biomarkers Prev, 2006. **15**(2): p. 381-4.

22. Clegg, D.O., et al., *Glucosamine, chondroitin sulfate, and the two in combination for painful knee osteoarthritis.* N Engl J Med, 2006. **354**(8): p. 795-808.

23. Anderson, J.W., R.J. Nicolosi, and J.F. Borzelleca, *Glucosamine effects in humans: a review of effects on glucose metabolism, side effects, safety considerations and efficacy.* Food Chem Toxicol, 2005. **43**(2): p. 187-201.

24. Jordan, K.M., et al., *EULAR Recommendations 2003: an evidence based approach to the management of knee osteoarthritis: Report of a Task Force of the Standing Committee for International Clinical Studies Including Therapeutic Trials (ESCISIT).* Ann Rheum Dis, 2003. **62**(12): p. 1145-55.
